# Pyramiding of multiple strong-culm genes originating from *indica* and tropical *japonica* to the temperate *japonica* rice

**DOI:** 10.1038/s41598-022-19768-3

**Published:** 2022-09-13

**Authors:** Taiichiro Ookawa, Tomohiro Nomura, Eri Kamahora, Mingjin Jiang, Yusuke Ochiai, Ahmad Fahim Samadi, Takuya Yamaguchi, Shunsuke Adachi, Keisuke Katsura, Takashi Motobayashi

**Affiliations:** 1 Graduate School of Agriculture, Tokyo University of Agriculture and Technology, 3-5-8 Saiwai-cho, Fuchu, Tokyo 183-8509 Japan; 2 Rice Research Institute of Guizhou Academy of Agricultural Science, Guiyang, 550006 Guizhou China; 3 Toyama Prefectural Tonami Agriculture and Forestry Promotion Center, 1-7 Saiwai-cho, Tonami, Toyama 939-1386 Japan

**Keywords:** Plant breeding, Plant genetics

## Abstract

Severe lodging has recurrently occurred at strong typhoon’s hitting in recent climate change. The identification of quantitative trait loci and their responsible genes associated with a strong culm and their pyramiding are important for developing high-yielding varieties with a superior lodging resistance. To evaluate the effects of four *strong-culm genes* on lodging resistance, the temperate *japonica* near isogenic line (NIL) with the introgressed *SCM1* or *SCM2* locus of the *indica* variety, Habataki and the other NIL with the introgeressed *SCM3* or *SCM4* locus of the tropical *japonica* variety, Chugoku 117 were developed. Then, we developed the pyramiding lines with double,triple and quadruple combinations derived from step-by-step crosses among NIL-SCM1–NIL-SCM4. Quadruple pyramiding line (NIL-SCM1 + 2 + 3 + 4) showed the largest culm diameter and the highest culm strength among the combinations and increased spikelet number due to the pleiotropic effects of these genes. Pyramiding of strong culm genes resulted in much increased culm thickness, culm strength and spikelet number due to their additive effect. *SCM1* mainly contributed to enhance their pyramiding effect. These results in this study suggest the importance of identifying the combinations of superior alleles of strong culm genes among natural variation and pyramiding these genes for improving high-yielding varieties with a superior lodging resistance.

## Introduction

The increase in global population is expected to accelerate food demand and the risk of famine^1^. In addition to population pressure, global warming has increased the intensity of super typhoons and their effects on rice production in the Philippines and other Asian regions^2,3^.

During the last century, the Green Revolution achieved an increase in rice production because the *semi-dwarf1* (*sd1*) gene was used to improve lodging resistance and yield^4^. However, despite the utilization of *sd1* for rice breeding, lodging has occurred in many semi-dwarf varieties when strong typhoons hit East and Southeast Asian countries. Thus, *sd1* alone is insufficient to avoid rice lodging under strong typhoon conditions^5^. To breed high-yielding varieties, it is necessary to develop new strategies to improve lodging resistance in rice. Instead of relying solely on *sd1*, alternative genes controlling culm strength are needed to develop superior lodging-resistant varieties.

In *japonica* varieties, *sd1* normally produces weak and fine culms that are ineffective in enhancing culm strength. Incorporating alternative strong-culm genes represents a new approach to improving lodging resistance and further increasing rice productivity^6^. However, rice breeders have encountered difficulties in improving strong-culm varieties because culm strength is a complex trait controlled by multiple genes.

Initially, we divided lodging resistance into component traits associated with strong culm. To breed high-yielding varieties, it is important to clarify the traits related to a high lodging resistance. For breaking-type lodging resistance, breaking strength of the basal culm is reflected by its bending moment at breaking (M). M is composed of a section modulus (SM), which represents culm thickness, and bending stress (BS), which indicates culm stiffness. SM is associated with outer diameter and culm wall thickness. In contrast, BS is associated with properties of the cell wall components, including lignin, cellulose, and hemicellulose. For bending-type lodging resistance, the bending strength of the culm is reflected by its Flexural rigidity (FR). FR is composed of a secondary moment of inertia (SMI), which indicates culm thickness, and the Young’s modulus (YM), which indicates culm plasticity. In a previous study, we found that large varietal differences in the M and FR of the basal culm were related to lodging resistance attributable to these component traits^7^.

To identify the QTLs and the genes responsible for lodging resistance, we chose *indica* type variety ‘Habataki’ and tropical *japonica* type ‘Chugoku 117’ as the donor parents, as these varieties exhibit thick culm and display large SM values compared to typical *japonica* varieties. From Habataki, an improved high-yielding *indica* variety with strong, thick culms, we identified QTLs associated with a thick culm: *SCM1* on chromosome 1 and *SCM2* on chromosome 6. *SCM1* included *Gn1a*-controlled grain number^8^, and *SCM2* was identical to *ABERRANT PANICLE ORGANIZATION1* (*APO1*), which encodes an F-box-containing protein involved in controlling the rachis branching number of panicle^6,9^. *SCM2*/*APO1* increased spikelet number and culm diameter due to the pleiotropic effects of this gene. From Chugoku 117, a tropical *japonica* type high-yielding line with strong, thick culms, we also identified QTLs associated with a thick culm: *SCM3* on chromosome 3 and *SCM4* on chromosome 2. *SCM3* was identified as being identical to rice *FINE CULM1* (*FC1*), which encodes a TCP domain-bearing transcription factor and controls tiller number through positively regulating strigolactone signaling^10^.

After identifying the QTLs, we developed the near-isogenic line NIL-SCM2 by introducing a 484-kb Habataki genome segment containing *SCM2*/*APO1* into the Koshihikari genetic background by repeated backcrossing. We also developed NIL-SCM3 by introducing a 163-kb Chugoku117 genome segment containing *SCM3*/*FC1* into the Koshihikari genetic background. Finally, we produced NIL-SCM1 and NIL-SCM4. SM and SMI of all four NILs exceeded those of Koshihikari, but were smaller than those of the donor parents, Habataki and Chugoku 117. This indicates that rice breeders are not able to develop superior lodging-resistant varieties by using only one strong-culm gene, because strong-culm traits are controlled by multiple genes.

To develop superior lodging-resistant varieties, we need to accumulate multiple QTLs for a strong culm. The QTL pyramiding method is used for accumulating beneficial QTLs by using Maker Assisted Selection (MAS)^11^. We established the hypothesis that pyramiding with the best combination of QTLs for strong culm would strengthen rice culms and achieve improved lodging resistance. In our previous study^10^, we crossed NIL-SCM3 with NIL-SCM2 to produce a double pyramiding line (NIL-SCM2 + 3). This pyramiding line exceeded both its parents and Koshihikari in terms of culm diameter, indicating the additive effect of the two QTLs. These results demonstrated that pyramiding QTLs for a strong culm improved lodging resistance in rice.

In this study, we further developed the pyramiding lines with double, triple, and quadruple NILs derived from step-by-step crosses among NIL-SCM1 (SCM1), NIL-SCM2 (SCM2), NIL-SCM3 (SCM3), and NIL-SCM4 (SCM4), and evaluated culm thickness, strength, and lodging resistance.

## Results

### Pyramiding effects of strong-culm genes on traits associated with lodging resistance

**Figure 1:**
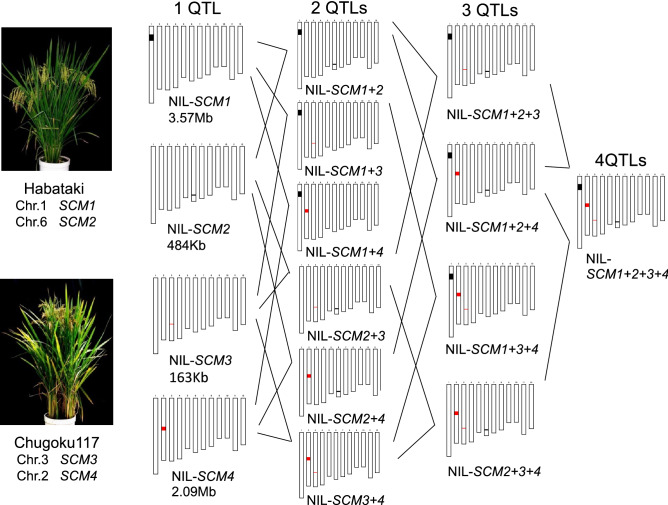
The breeding process for the pyramiding lines. Pyramiding lines with double NILs (SCM1 + 2, SCM1 + 3, SCM1 + 4, SCM2 + 4, SCM2 + 3, SCM3 + 4), triple NILs (SCM1 + 2 + 4, SCM1 + 2 + 3, SCM1 + 3 + 4, SCM2 + 3 + 4), and a quadruple NIL (SCM1 + 2 + 3 + 4) were developed. These combinations were derived from step-by-step crosses among NIL-SCM1–NIL-SCM4. Habataki segments of 3.57 Mb and 484 kb were introgressed in NIL-SCM1 and NIL-SCM2 of the Koshihikari genetic background. Chugoku 117 segments of 163 kb and 2.09 Mb were introgressed in NIL-SCM3 and NIL-SCM4 of the Koshihikari genetic background.

**Figure 2:**
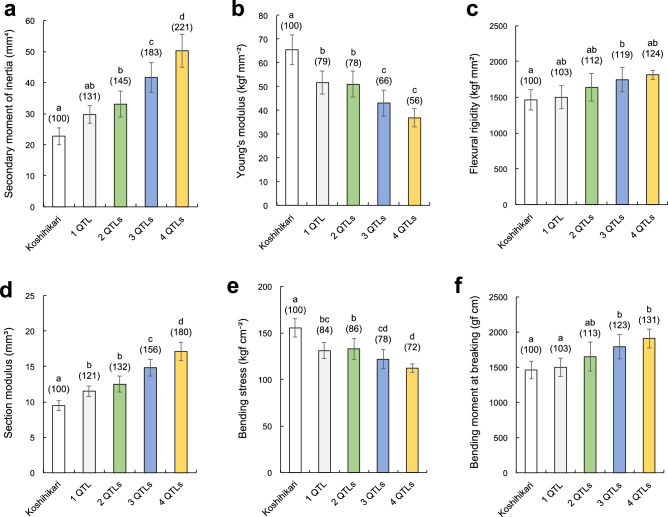
Comparison of strong-culm traits related to lodging resistance by number of QTL pyramiding. (**a**–**c**) Comparison of bending-type lodging resistance-related traits. (**a**) Secondary moment of inertia. (**b**) Young's modulus. (**c**) Flexural rigidity. (**d**–**f**) Comparison of breaking-type lodging resistance-related traits. (**d**) Section modulus. (**e**) Bending stress. (**f**) Bending moment at breaking. Each column shows mean ± SD of three replicates. Different letters indicate significant differences between lines at the 5% level (Tukey’s test). The numbers in parentheses show values relative to Koshihikari (100).

Figure 1 shows the breeding process for the pyramiding lines. Pyramiding lines with double NILs (SCM1 + 2, SCM1 + 3, SCM1 + 4, SCM2 + 3, SCM2 + 4, SCM3 + 4), triple NILs (SCM1 + 2 + 3, SCM1 + 2 + 4, SCM1 + 3 + 4, SCM2 + 3 + 4), and a quadruple NIL (SCM1 + 2 + 3 + 4) were developed. To investigate the effect of QTL pyramiding on strong-culm related traits, we compared the number of QTL pyramiding for each trait related to bending-type lodging resistance using single NILs, pyramiding lines and Koshihikari (Fig. 2). The mean Flexural rigidity (FR) was higher in double, triple, and quadruple pyramiding lines, compared to single NILs and Koshihikari, and increased proportionally to the number of QTL pyramiding (Fig. 2c). For the component traits of FR, SMI increased with QTL pyramiding number, while the Young’s modulus decreased (Fig. 2a,b).

Figure 2d–f shows the M and its component traits, SM and BS, which are indicators of breaking-type lodging resistance. The mean M in the triple and quadruple pyramiding lines was significantly higher than in Koshihikari. For the component traits, the cross-section modulus increased with the QTL pyramiding number, while the BS decreased.

**Figure 3:**
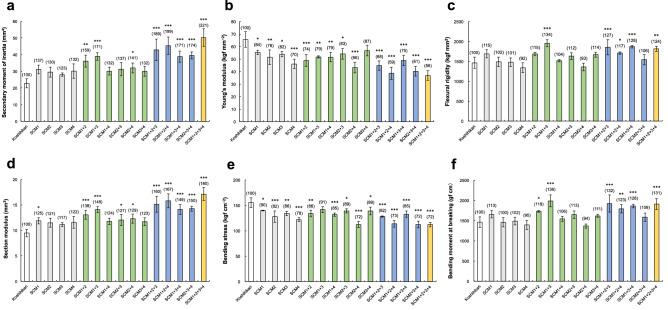
Comparison of strong-culm traits related to lodging resistance for each QTL combination. (**a**–**c**) Comparison of bending-type lodging resistance-related traits. (**a**) Secondary moment of inertia. (**b**) Young's modulus. (**c**) Bending rigidity. (**d**–**f**) Comparison of breaking-type lodging resistance-related traits. (**d**) Section modulus. (**e**) Bending stress. (**f**) Bending moment at breaking. Each column shows mean ± SD of three replicates. *, ** and *** indicate significant differences compared to Koshihikari at the 5%, 1% and 0.1% level (Dunnett's test). The numbers in parentheses show the values relative to Koshihikari (100).

For each of these traits, we examined which combination of QTLs had the highest pyramiding effect on the lodging resistance-related traits (Fig. 3). Figure 3A–C shows the bending-type lodging resistance-related traits for each combination of QTLs. In the double pyramiding lines, the SMI of SCM1 + 2, SCM1 + 3, and SCM2 + 4 was significantly increased compared with Koshihikari, most notably in SCM1 + 2 and SCM1 + 3. In the triple pyramiding lines, the pyramiding effect was significantly higher in all three lines than in Koshihikari, especially in SCM1 + 2 + 3 and SCM1 + 2 + 4 (Fig. 3c). The Young’s modulus of the almost all pyramiding lines was significantly reduced compared with Koshihikari, with only SCM3 + 4 not being significantly different. Among the double pyramiding lines, SCM2 + 4 had the lowest Young’s modulus. In the triple pyramiding lines, Young’s modulus was highest in SCM1 + 3 + 4 and lowest in SCM1 + 2 + 4 (Fig. 3b).

Figure 3D–F shows the breaking-type lodging resistance-related traits for each combination of QTLs. The SM of SCM1 + 2, SCM1 + 3, SCM2 + 3, and SCM2 + 4 was significantly increased compared with Koshihikari, and the pyramiding effect of SCM1 + 2 and SCM1 + 3 was particularly high in the double pyramiding lines. In the triple pyramiding lines, SM was significantly increased in all lines, and the pyramiding effect was particularly high in SCM1 + 2 + 3 and SCM1 + 3 + 4 compared with Koshihikari. Bending stress was significantly reduced in almost all lines compared to Koshihikari, with only SCM1 + 3 not being significantly different. Among the double pyramiding lines, SCM2 + 4 had the lowest BS. In the triple pyramiding lines, BS showed the same trend as observed with Young’s modulus. Among the double pyramiding lines, the M of SCM1 + 2 and SCM1 + 3 was significantly increased compared with Koshihikari. Among the triple pyramiding lines, M was significantly increased compared with Koshihikari in all lines except SCM2 + 3 + 4.

**Figure 4:**
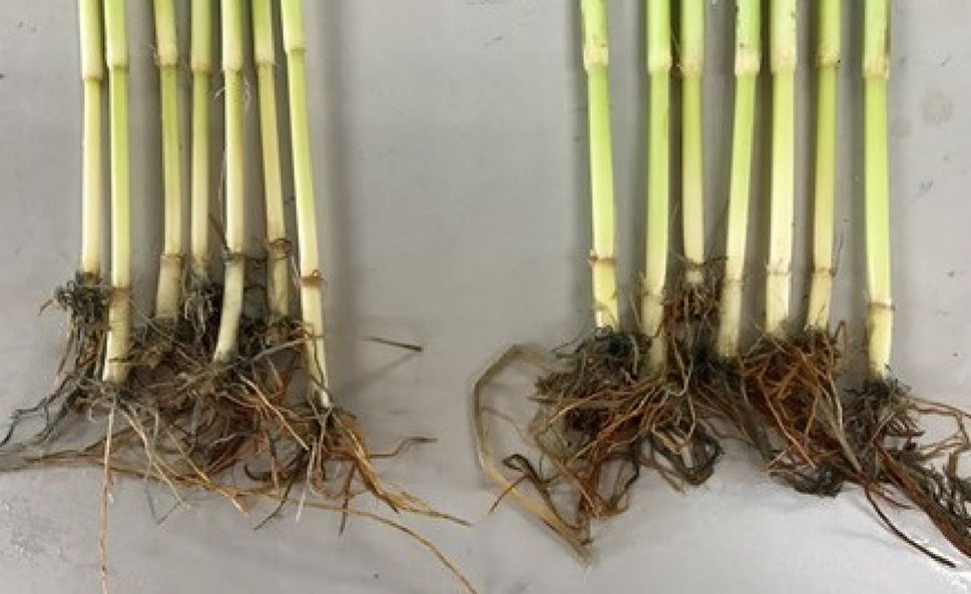
Comparison of the lowest elongating internodes between Koshihikari and SCM1 + 2 + 3 + 4. Representative photographs of the lowest elongating internodes in Koshihikari (left) and SCM1 + 2 + 3 + 4 (right).

Figure 4 shows a photograph of representative main culms in the quadruple pyramiding line SCM1 + 2 + 3 + 4 and Koshihikari. It can be seen that SCM1 + 2 + 3 + 4 had a thick culm phenotype compared with Koshihikari.

### Additional effects and epistatic interactions for strong-culm genes

Multiple linear regression analysis was then used to examine the contribution of each QTL for strong-culm traits and epistasis among QTLs in single NILs and pyramiding lines.

Table S1 shows the contribution of each QTL and the estimated interactions between QTLs in SMI and SM. The coefficients and t-values increased in the order of SCM1, SCM2, SCM3, and SCM4 in SMI, indicating that the two QTLs from Habataki had a larger incremental effect on SMI. Moreover, the coefficients and t-values increased in the order of SCM1, SCM2, SCM3, and SCM4 in SM, indicating a larger contribution to SM. This was similar to the results observed for SMI. Regarding the interaction, a positive effect of epistasis was detected between SCM2 and SCM4 in both SM and SMI (*P* = 0.030), but the t-value was lower than any single QTL.

Figure S1 shows the relationship between the estimated and measured SMI and SM excluding interactions for Koshihikari and the pyramiding lines. If the trait values of the pyramiding lines could be accurately estimated using only the additive effects of QTLs, excluding epistasis, the measured and estimated values for each line would be equal and plot on a straight line with y = x. For SMI, the regression equation was y = 0.922 x + 4.564 with a coefficient of determination of 0.850. For SM, the regression equation was y = 0.995 x + 0.684 with a coefficient of determination of 0.873. The close proximity of y = x for both thick culm traits, and the high coefficients of determination indicated that estimates of the individual QTLs, excluding interactions among QTLs, explained the majority of the thick culm traits in the pyramiding lines. These results indicated that culm thickness was largely determined by simple additive effects that the contribution of epistasis is not highly involved.

### Evaluation of the pyramiding effects of strong-culm genes on lodging resistance

To evaluate the effect of the pyramiding of strong-culm QTLs on lodging resistance, we conducted an artificial typhoon test and measured the degree of lodging (Fig. S2). The artificial typhoon test was performed on Koshihikari and three pyramiding lines (SCM1 + 2 + 3, SCM1 + 3 + 4, and SCM1 + 2 + 3 + 4), which were more effective in QTL pyramiding on strong-culm associated traits. Compared with Koshihikari, only a small degree of curvature in the pyramiding lines was observed (Fig. S2a). Thus, the angle of curvature of the pyramiding lines was investigated. The results are shown in Figure S2b. Among the four lines tested, Koshihikari had the largest bending angle (43°) after the artificial typhoon test. In contrast, the three pyramiding lines had a smaller angle of bending than Koshihikari. In particular, the bending of SCM1 + 3 + 4 was significantly reduced by 58% compared to Koshihikari.

**Figure 5:**
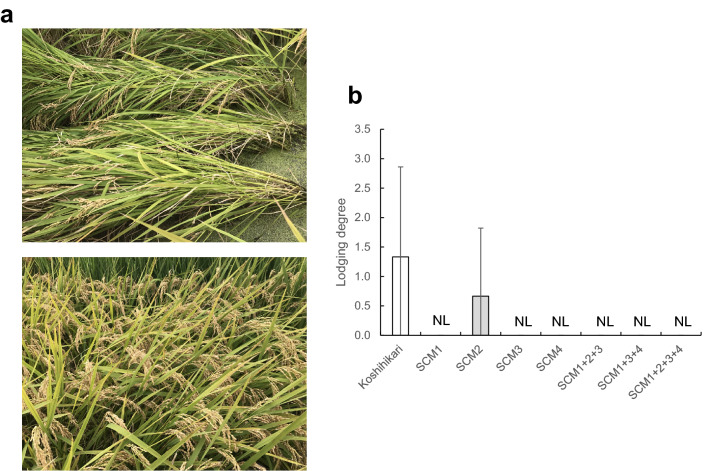
Evaluation of lodging resistance in paddy fields during harvest. (**a**) Representative photographs of Koshihikari (top) and SCM1 + 2 + 3 + 4 (bottom) at the 35 days after heading. (**b**) Degree of lodging. NL: No lodging.

To evaluate the occurrence of lodging in actual paddy fields, we measured the degree of lodging (Fig. 5). Figure 5a shows representative photographs of Koshihikari and SCM1 + 2 + 3 + 4 at the late maturing stage. In Koshihikari, lodging occurred after typhoon exposure, whereas no lodging occurred in SCM1 + 2 + 3 + 4. The degree of lodging was greater in Koshihikari due to the high degree of plant bending; however, no lodging occurred in the other lines, except for SCM2 (Fig. 5b).

### Causal factors of strong culm in pyramiding lines

**Figure 6:**
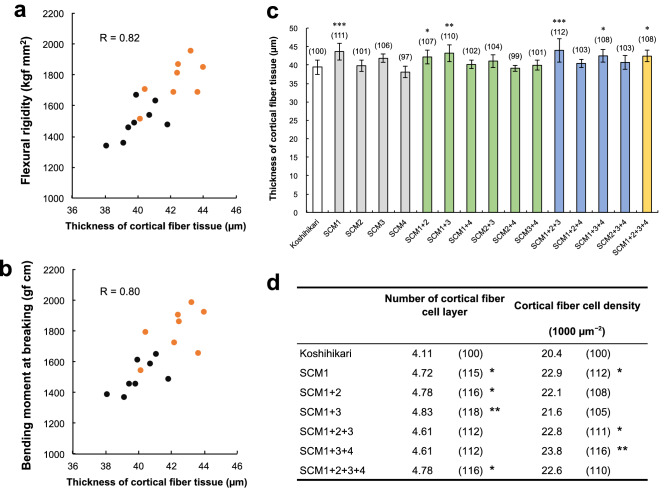
The relationship between culm strength and cortical fiber tissue thickness, and a comparison of structural properties in cortical fiber tissue among pyramiding lines. (**a**) The relationship between Flexural rigidity and cortical thickness. Orange circles indicate the lines including SCM1. (**b**) The relationship between bending moment at breaking and cortical thickness. The numbers in parentheses show values relative to Koshihikari (100). (**c**) Comparison of cortical fiber tissue thickness in Koshihikari and pyramiding lines. (**d**) The number of cortical fiber cell layers and cortical fiber cell density in Koshihikari and pyramiding lines. The numbers in parentheses show the values relative to Koshihikari (100). *, ** and *** indicate significant differences compared to Koshihikari at the 5%, 1% and 0.1% level (Dunnett's test).

To investigate the causal factors of high FR and M in the pyramiding lines, we focused on the structural properties in cortical fiber tissue (Fig. 6). The correlation coefficient between FR and the thickness of the cortical fiber tissue was 0.82, and the correlation coefficient between M and the thickness of the cortical fiber tissue was 0.80. In both cases, a strong positive correlation was observed (Fig. 6a,b). The lines containing SCM1 exhibited thicker cortical fiber tissue, large FR and M. The lines with a significantly thicker cortical fiber tissue compared to Koshihikari were SCM1, SCM1 + 2, SCM1 + 3, SCM1 + 2 + 3, SCM1 + 3 + 4, and SCM1 + 2 + 3 + 4, all of which contained SCM1 (Fig. 6c). Figure 6d shows the number and density of cell layers in the cortical fiber tissue of lines containing SCM1. The number of cell layers was significantly increased in SCM1, SCM1 + 2, SCM1 + 3, and SCM1 + 2 + 3 + 4, and the cell density was significantly increased in SCM1, SCM1 + 2 + 3, and SCM1 + 3 + 4 compared to Koshihikari.

### Pleiotropic pyramiding effects of strong-culm genes on traits associated with yield components

To investigate the effects of strong-culm QTL pyramiding on the pleiotropic expression of yield component traits, we compared the yield and yield components among Koshihikari and pyramiding lines with a superior lodging resistance (Table S2). In the single NILs (SCM1, SCM3, and SCM4), the panicle number tended to decrease compared to Koshihikari in both years, especially in SCM1 and SCM3 in 2018, with 19% and 17% decreases in panicle number, respectively. In the pyramiding lines, the panicle number of SCM1 + 2 + 3, SCM1 + 3 + 4, and SCM1 + 2 + 3 + 4 was lower than that of Koshihikari. In SCM1 + 2 + 3 and SCM1 + 2 + 3 + 4, panicle number decreased significantly. In particular, the panicle number in SCM1 + 2 + 3 + 4 decreased significantly in both years, 31% in 2017 and 44% in 2018, compared with that in Koshihikari. On the other hand, the grain number per panicle increased in the four single NILs. Notably, the grain number of SCM1 significantly increased by 36% in 2017 and 35% in 2018 compared with that of Koshihikari. The grain number per panicle of the pyramiding lines was significantly higher than that of Koshihikari in both years, and this increase was greater than those of the single NILs. In particular, the grain number per panicle in SCM1 + 2 + 3 + 4 increased by 61% in 2017 and 68% in 2018 compared to Koshihikari. Although large differences in the yield components were found among Koshihikari and pyramiding lines, there were no differences in grain yield between Koshihikari and the pyramiding lines.

Finally, we investigated the eating quality in the pyramiding line SCM1 + 3 + 4 (Table S3). SCM1 + 3 + 4 had a premium eating quality with a low protein, which is similar to Koshihikari. Strong culm genes of pyramiding lines did not affect to their eating quality in the Koshihikari genetic background. We released the new variety ‘Sakura prince’ with a strong culm derived from SCM 1 + 3 + 4 using marker assisted selection in 2022.

## Discussion

In this study, we used donor varieties to develop NILs with strong culm in rice. Habataki is the *indica* type variety with *SCM1* and *SCM2*^6^. To improve lodging-resistant *japonica* varieties, NIL-SCM1 and NIL-SCM2 were developed by introducing Habataki genome segments into the leading *japonica* variety, Koshihikari. *SCM2* is identical to the *APO1* gene, and the *indica* type Habataki has a superior gain-of-function allele for *APO1*. Jang et al.^12^ reported that the *APO1-3* haplotype, which is identical to *SCM2*, was detected only in many *indica* and Tongil type accessions. Tongil type varieties were derived from crosses between *indica* and *japonica* varieties developed in Korea. Habataki was also derived from Tongil type Korean varieties. Habataki had the same haplotype, therefore the superior allele of *SCM2* in Habataki was introduced from Korean Tongil type varieties. While the causal gene associated with *SCM1* has not been identified, the candidate genome region on chromosome 1 contains *Gn1a*. A loss-of-function allele for *Gn1a* was observed to increase grain number per panicle in Habataki^8^. *Gn1a* affects not only grain number but also culm thickness, because NIL-Gn1a with a narrow Habataki genome segment has thick culms compared with Koshihikari. It has been reported that the null *gn1a* improved lodging resistance through increasing the culm diameter^13^.

The donor variety Chugoku 117 is the *tropical japonica* type variety with *SCM3* and *SCM4*^10^. *SCM3* was isolated as an effective QTL, and the causal gene is identical to *FC1* in rice^14^. This gene has been reported to positively control strigolactone signaling. *FC1* suppresses tiller number by enhancing gene expression*. SCM3*, a gain-of-function allele of *FC1* also contributes to increase spikelet number per panicle despite the decreased tiller number. Substitution lines also have a positive role in enhancing culm diameter and spikelet number per panicle.

The effects of *SCM* genes on morphological traits related to tillering, such as culm length and panicle length, were investigated. The results showed that the culm length and ear length of the four independent NIL lines were similar to those of Koshihikari; therefore, these *SCM* genes did not significant alter the morphological traits (Table S4). More than 90% of the area of a culm is composed of parenchyma cells^15^, and there are two possible reasons for the increase in culm diameter: an increase in parenchymal cell number or an increase in cell area. Our previous reports clarified that *SCM2* and *SCM4* increase the number of parenchymal cells in the transverse section of culm, and *SCM3* increases the number of parenchymal cells and cell area^6,10^. It is inferred that culm diameter increased due to an increase in parenchymal cell number and the expansion of cell area, as observed in other QTLs.

To evaluate the pyramiding effects of the *SCM* genes, we developed pyramiding lines with double, triple and quadruple NILs. SM and SMI associated with a culm diameter increased proportionally to the number of pyramided SCM genes. As a result, M and FR in quadruple NIL were the greatest among the pyramiding lines. This is the first report of a pyramiding effect associated with multiple strong-culm genes originating from different donor varieties in marker assisted breeding of lodging resistance.

The pyramiding lines with *SCM1* had large SMI and YM, indicating the pleiotropic effect of *SCM1*. These lines also had a thick, developed culm cortical fiber tissue. The cortical fiber tissue in rice is located in the outer layer of culm, and contributes to increases in strength^5,15^. *Gn1a* located in *SCM1* regulates cytokinin metabolism^8^. Since cytokinin promotes cell division, *Gn1a* increases the spikelet number per panicle and culm diameter. Cytokinins may also be involved in cortical fiber tissue thickening.

We analyzed additional effects and epistatic interactions for *SCMs* using multiple regression analysis. Regression analysis indicated that the cumulative effects of SCMs without significant epistatic interactions largely explained the phenotypic traits. The molecular mechanism underlying the pyramiding effect of *SCM2* and *SCM3* using NIL-SCM2 + 3 on culm diameter were investigated^10^. This study indicates that the mechanism of pyramiding effect, at least in part, involves regulation of the strigolactone and *APO1* signaling pathways, with each signaling mechanism controlling meristem size associated with culm diameter, grain number and/or panicle number, independently. *APO1* encodes an F-box protein and plays a role in the temporal regulation of meristem fate. *FC1* encodes a TCP domain-bearing transcription factor and controls tiller number through positively regulating strigolactone signaling. These genes are thought to be independently regulated because they belong to different categories associated with the morphogenesis of the panicle and tiller. Li et al.^16^ reviewed the genetic regulatory network of panicle architecture in rice, and divided *APO1* and *Gn1a* into different regulatory networks controlling panicle branch and spikelet formation in rice. *Gn1a* encodes cytokinin oxidase and affects cytokinin levels and the production of primary branches in panicle. In this study, strong-culm genes elicit pleiotropic effects on panicle size and culm thickness, therefore these genes dominate independent regulatory networks, and might produce additional effects on the phenotypes of panicle and culm.

We also investigated the pleiotropic effects of strong-culm gene pyramiding on the traits associated with panicle size and yield components. Pyramiding of *SCMs* had no effect on culm length, but some combinations of QTLs increased ear length (Table S4). The panicle length in single NILs was almost identical to Koshihikari, while the panicle length of the pyramiding lines was significantly greater than that of Koshihikari, suggesting that panicle length increases additively by pyramiding depending on the QTL combination. Spikelet number per panicle in triple and quadruple NILs was greater than in Koshihikari and single NILs. The spikelet number per panicle is mainly controlled by a range of panicle branching such as *APO1* and *Gn1a*, and is also associated with tillering genes such as *FC1*^8–10^. Zeng et al.^17^ successfully developed a “super-rice” using rational design. They combined many beneficial alleles for yield and quality (including *Gn1a* and *APO1*) by marker assisted crossing among *indica* and *japonica* type varieties and developed new pyramiding lines. In this study, the pyramiding of other strong-culm genes, *SCM3* and *SCM4* with these two genes had an additional effect on the spikelet number per panicle.

Pyramiding of strong-culm genes had negative effects on panicle number per plant. Yano et al.^10^ pointed out that *FC1* allele derived from the tropical *japonica* type Chugoku 117 with a thick culm has a negative effect on panicle number. Cui et al.^18^ reported that CRISPR-Cas9 could be used to easily produce alleles with an optimum expression level for *FC1*, and suggested that breeders could isolate and utilize desirable alleles with more appropriate levels of expression for optimizing breeding targets. Further studies will be needed for regulating the optimum levels of gene expression associated with the panicle and tiller formations in meristem and for searching the superior combination of pyramiding.

In this study, multiple chromosome segments with strong-culm genes were introgressed in the leading temperate *japonica* variety ‘Koshihikari’ with a high quality, which has a fine culm, resulting in the development of a new variety with a thick culm named ‘Sakura prince’. This study highlights the importance of identifying combinations of superior alleles of strong-culm genes among natural variation, and pyramiding these genes to improve high-yielding or high-quality varieties with superior lodging resistance. The results of this study in rice can be applied to improve lodging resistance in other gramineous crops. The stem lodging resistance is also important for developing high yielding varieties in gramineous crops such as wheat and barley^19^. *APO1* and *FC1* (*OsTB1*), the strong culm genes of rice, are also pleiotropically related to the number of tillers and the number of spikelets per panicle. *TaAPO-A1*^20^ and *TaTB1*^21^, which are homologous to these genes, have been reported to be involved in panicle morphogenesis as in rice. Therefore, it may be possible to accumulate strong culm genes in wheat by pyramiding genes such as *TaAPO-A1* and *TaTB1*. It will be expected that the pyramiding effect of strong culm genes in other gramineous crops also improves their lodging resistance.

## Methods

### Plant materials

All near isogenic lines with a thick culm, NIL-SCM1, NIL-SCM2, NIL-SCM3, and NIL-SCM4, and the pyramiding lines were developed by MAS.

The near isogenic line (NIL) of the temperate *japonica* type variety ‘Koshihikari’ with introgressed *SCM1* or *SCM2* locus of the *indica* type ‘Habataki’ (NIL-SCM1, NIL-SCM2)^6^ and the other Koshihikari NIL with the introgressed *SCM3* or *SCM4* locus of tropical *japonica* type ‘Chugoku 117’ (NIL-SCM3, NIL-SCM4)^10^ were developed as described in previous reports.

To evaluate the pyramiding effects of these QTLs, we developed pyramiding lines with double NILs (SCM1 + 2, SCM1 + 3, SCM1 + 4, SCM2 + 4, SCM2 + 3, SCM3 + 4), triple NILs (SCM1 + 2 + 3, SCM1 + 2 + 4, SCM1 + 3 + 4, SCM2 + 3 + 4), and a quadruple NIL (SCM1 + 2 + 3 + 4) using marker assisted selection. Habataki segments of 3.57 Mb (RM10286–RM10539) and 484 kb (RM20545–RM20572) were introgressed in NIL-SCM1 and NIL-SCM2 of the Koshihikari genetic background. Chugoku 117 segments of 163 kb (RM15761–RM15772) and 2.09 Mb (RM13228–RM3858) were introgressed in NIL-SCM3 and NIL-SCM4 of the Koshihikari genetic background. The pyramiding lines were derived from step-by-step crosses among NIL-SCM1–NIL-SCM4.

### Plant cultivation for phenotyping

Field experiments for the evaluation of lodging resistance in pyramiding lines were conducted on the Experimental Farm of the Field Science Center, Tokyo University of Agriculture and Technology in 2017–2018. Because growth conditions were similar during these two years, only the cultivation methods for 2018 are described here.

Seeds were sown in nursery boxes on May 8, 2018. Seedlings at the fifth-leaf stage were transplanted to a paddy field on Tama River alluvial soil at a rate of one plant per hill on May 25, 2017. Planting density was 22.2 hills m^−2^, at a spacing of 15 cm × 30 cm. Chemical fertilizers were applied as a basal dressing at a rate of 50, 60, and 60 kg each of N, P, and K per ha, respectively. The field was kept under submerged conditions throughout the course of the experiments. The experiment was designed with three randomly arranged replicates (1.8 m^2^ for phenotyping of lodging resistance and 2.25 m^2^ for yield survey per replicate).

### Evaluation of lodging resistance traits

The physical parameters of the main culm, which are closely associated with lodging resistance, were precisely evaluated. The main culms were sampled from plants showing average leaf number, and the bending load at breaking was measured at a distance of 4 cm between two supporting points using a universal testing machine (Tensilon RTG-1210; A&D, Tokyo, Japan) according to the method of Ookawa and Ishihara^7^.

In breaking-type lodging resistance, bending moment of the basal internode at breaking (M) describes the resistance to breaking. M is the product of section modulus (SM) and bending stress (BS). M and SM were calculated using the following formulas:

1 $${\text{M}} = {\text{SM }} \times {\text{BS}}$$

2 $${\text{SM}} = \pi /32 \times ({\text{a}}_{1}^{3} {\text{b}}_{1} - {\text{a}}_{2}^{3} {\text{b}}_{2} )/{\text{a}}_{1}$$

a_1_, short outer-diameter; b_1_, long outer-diameter; a_2_, short inner-diameter; b_2_, long inner-diameter

In bending-type lodging resistance, flexural rigidity (FR) describes the resistance to bending. FR is the product of Young’s modulus (YM) and the secondary moment of inertia (SMI). SMI and FR were calculated using the following formulas:

3 $${\text{SMI}} = \pi /64 \times ({\text{a}}_{1}^{3} {\text{b}}_{1} - {\text{a}}_{2}^{3} {\text{b}}_{2} )$$

4 $${\text{FR}} = {\text{YM}} \times {\text{SMI}}$$

### Artificial typhoon test

The artificial typhoon test was performed at 20 days after heading. Plants with an average number of stems were used for the measurements. The plants were dug up, the soil at the roots was removed with water and the base of the plant was fixed with a clamp. A large fan with a shower was used to generate a windstorm of 15 m s^−1^ for 1 min, and the angle of curvature of the plant from the vertical at 20 cm above the root-shoot transition was measured with a multi-level A-300 (Shinwa Rules, Niigata, Japan).

### Yield survey and eating quality

The plants were dug up at harvest time, and the soil was removed with water and allowed to air dry in a plastic greenhouse. The panicles were cut at the neck of panicle, and the plants with average panicle weight and number were selected for the measurement of yield components. After removing the roots with scissors, the dry matter weight of the stems and leaves was measured, and the yield index was calculated from the panicle weight and the dry matter weight of the stems and leaves. After the grains were hulled using testing husker (THU35B; Satake, Hiroshima, Japan), they were shaken 10 times with a 1.8 mm sieve and sorted into brown rice and crushed rice. The number of brown rice was measured with an automatic counter (KC-10 M; Fujiwara Scientific, Tokyo, Japan), and the percentage of ripened grains and 1000-grain weight were calculated. The brown rice was dried at 80 °C for 72 h and the dry weight was measured. 1000-grain weight of brown rice was determined at 15% moisture content.

Taste score (Mido score) of cooked rice samples was measured using a Toyo Mido Meter (MB-90A; Wakayama, Japan). The sensory properties of cooked rice palatability were evaluated by 20 trained panels in the Toyama Agricultural, Forestry & Fisheries Research Center. This test involved scoring the cooked rice quality on the scales from − 2 to + 2.

### Maker assisted selection of pyramiding lines

The resultant informative single sequence repeat markers (SSRs) were used for genotyping of progenies derived from crosses between NILs.

Genomic DNA was extracted from leaves using the CTAB method^22^ and pure DNA samples were used for SSR genotyping^23^. For polymerase chain reaction (PCR) of SSRs, a 1 µL aliquot of DNA extract was used as the template for PCR amplification on a GeneAmp 9700 thermocycler (Applied Biosystems, California, USA). The 10 µL reaction volume contained 1 µL template DNA, 1 µL of 10 × PCR buffer, 25 mM of MgCl_2_, 2 mM of each dNTP, 2 µL 50% glycerol, 0.1 µL *Taq* DNA polymerase (5 U µL^−1^), 0.2 µL of a 20 pM solution of each primer, and 3.7 µL of H_2_O. Amplification was performed for 30 cycles (30 s at 94 °C, 1 min at 60 °C and 1 min at 72 °C) followed by 7 min at 72 °C. The amplified products were electrophoresed in an agarose gel to detect polymorphisms.

### Statistical analysis

Tukey’s test or Dunnett’s test was performed using R software^24^ for comparison between lines. In Dunnett’s test, Koshihikari was used as the control group. Multivariable linear regression (MLR) model was performed using JMP software ver12.0 (JMP Statistical Discovery) to quantify the additive effect of each QTL and epistasis between QTLs as follows^25^:

5 $$\begin{aligned} Y & = \beta_{0} + \beta_{1} {\text{SCM}}1 \, + \beta_{2} {\text{SCM}}2 \, + \beta_{3} {\text{SCM}}3 \, + \beta_{4} {\text{SCM }}4 \, + \beta_{5} {\text{SCM}}1 \times {\text{SCM}}2 \\ & \quad + {\kern 1pt}...+ \beta_{15} {\text{SCM}}1 \, \times {\text{ SCM}}2 \, \times {\text{ SCM}}3 \, \times {\text{ SCM}}4 \, + \varepsilon \\ \end{aligned}$$

where *Y* is the predicted variable, *β*_0,_ *β*_2_ … *β*_15_ are the regression coefficient, SCM1, SCM2 SCM3, SCM4 are variables of each QTL, and *ε* is the stochastic error term of the regression. We also performed MLR to quantify the additive effect of each QTL without epistatic effect (additive model). Then we estimated the phenotypic values of the pyramiding lines from the additive model.

## Statement

We have been granted permission to conduct all experiments using the rice varieties in this study, and we have followed national guidelines and regulations.

## Supplementary Information

Supplementary Information.

## Data Availability

All data generated or analyzed during this study are included in this published article and its supplementary information files.

## Abbreviations

BM: Bending moment at breaking
BS: Bending stress
FR: Flexural rigidity
NIL: Near isogenic line
QTL: Quantitative trait loci
SM: Section modulus
SMI: Secondary moment of inertia
YM: Young’s modulus
