# Supplementary material for: Pyramiding of multiple strong-culm genes originating from indica and tropical japonica to the temperate japonica rice
Source: Sci Rep. 2022 Sep 13;12:15400. doi: 10.1038/s41598-022-19768-3 (PMC9470567; doi:10.1038/s41598-022-19768-3)
Supplement: Supplementary file 1 — Supplementary Information. [file 41598_2022_19768_MOESM1_ESM.pdf]

Supplementary Information

Pyramiding of multiple strong-culm genes originating from *indica* and tropical *japonica* to the temperate *japonica* rice

Table S1. The contributions of QTLs and their interactions in secondary moment of inertia and section modulus.

	Secondary moment of inertia					Section modulus				
	**Coefficient**	**SE**	**t-value**	**p-value**		**Coefficient**	**SE**	**t-value**	**p-value**	
Intercept	21.94	1.18	18.64	<.0001	***	9.37	0.31	30.68	<.0001	***
SCM1	8.79	1.05	8.34	<.0001	***	2.37	0.27	8.68	<.0001	***
SCM2	7.15	1.05	6.79	<.0001	***	1.93	0.27	7.05	<.0001	***
SCM3	5.33	1.05	5.06	<.0001	***	1.53	0.27	5.59	<.0001	***
SCM4	4.44	1.05	4.22	0.0002	***	1.29	0.27	4.74	<.0001	***
SCM1×SCM2	3.56	2.11	1.69	0.101		0.77	0.55	1.42	0.166	
SCM1×SCM3	3.49	2.11	1.66	0.107		0.91	0.55	1.66	0.107	
SCM1×SCM4	-1.17	2.11	-0.56	0.581		-0.27	0.55	-0.49	0.626	
SCM2×SCM3	-0.19	2.11	-0.09	0.929		-0.09	0.55	-0.17	0.867	
SCM2×SCM4	4.78	2.11	2.27	0.030	*	1.30	0.55	2.37	0.024	*
SCM3×SCM4	-0.12	2.11	-0.06	0.956		-0.18	0.55	-0.33	0.745	
SCM1×SCM2×SCM3	-4.48	4.21	-1.06	0.296		-0.96	1.09	-0.87	0.389	
SCM1×SCM2×SCM4	8.19	4.21	1.94	0.061		2.20	1.09	2.02	0.052	
SCM1×SCM3×SCM4	-0.66	4.21	-0.16	0.876		-0.28	1.09	-0.25	0.803	
SCM2×SCM3×SCM4	4.24	4.21	1.01	0.321		1.02	1.09	0.94	0.357	
SCM1×SCM2×SCM3×SCM4	-13.99	8.42	-1.66	0.107		-3.88	2.18	-1.78	0.085	

*,** and *** indicate significant differences at 5%, 1% and 0.1% levels.

Table S2. Yield components, yield and harvest index.

		Panicle number (m^{-2})			Number of grains per panicle			Percentage of ripened grains (%)			1000-grain weight (g)			Grain yield ($t\ ha^{-1}$)			Harvest index		
2017	Koshihikari	392.6	(100)	a	101.2	(100)	a	75.7	(100)	ab	22.0	(100)	abc	6.59	(100)	a	35.9	(100)	a
	SCM1	318.5	(81)	ab	138.1	(136)	c	66.3	(88)	b	21.4	(97)	a	6.21	(94)	a	33.9	(95)	a
	SCM2	395.1	(101)	a	111.1	(110)	ab	68.6	(91)	ab	21.4	(97)	a	6.38	(97)	a	35.9	(100)	a
	SCM3	325.9	(83)	ab	109.3	(108)	ab	77.3	(102)	a	22.7	(103)	bc	6.22	(94)	a	35.5	(99)	a
	SCM4	348.1	(89)	ab	115.5	(114)	ab	77.4	(102)	a	21.2	(97)	a	6.58	(100)	a	36.4	(101)	a
	SCM1+2+3	284.0	(72)	b	143.9	(142)	cd	72.3	(96)	ab	21.1	(96)	a	6.21	(94)	a	37.1	(103)	a
	SCM1+3+4	312.3	(80)	ab	126.7	(125)	bc	71.7	(95)	ab	23.1	(105)	c	6.56	(100)	a	35.1	(98)	a
	SCM1+2+3+4	270.4	(69)	b	163.1	(161)	d	68.6	(91)	ab	21.6	(98)	ab	6.53	(99)	a	36.5	(102)	a
2018	Koshihikari	437.0	(100)	a	96.0	(100)	a	68.7	(100)	a	23.4	(100)	ab	6.73	(100)	a	32.5	(100)	a
	SCM1	351.9	(81)	abc	129.8	(135)	bc	67.0	(98)	a	21.1	(90)	d	6.36	(94)	a	34.0	(105)	a
	SCM2	381.5	(87)	ab	109.9	(114)	ab	65.9	(96)	a	20.6	(88)	d	5.71	(85)	a	32.3	(99)	a
	SCM3	363.0	(83)	abc	99.9	(104)	ab	71.3	(104)	a	21.8	(93)	cd	5.57	(83)	a	31.0	(95)	a
	SCM4	385.2	(88)	a	101.2	(105)	ab	69.1	(101)	a	20.8	(89)	d	5.59	(83)	a	32.7	(101)	a
	SCM1+2+3	281.5	(64)	bcd	155.7	(162)	cd	64.7	(94)	a	22.9	(98)	abc	6.48	(96)	a	33.2	(102)	a
	SCM1+3+4	277.8	(64)	cd	156.8	(163)	cd	65.9	(96)	a	23.8	(102)	a	6.78	(101)	a	35.1	(108)	a
	SCM1+2+3+4	244.4	(56)	d	161.3	(168)	d	64.2	(94)	a	22.3	(95)	bc	5.68	(84)	a	35.3	(108)	a

Data represents the mean of three replicates. Different letters indicate significant differences among lines at the 5% level (Tukey's test). The numbers in parentheses show the values relative to Koshihikari (100).

Table S3. The eating quality of the new developed variety ‘Sakura prince’.

	Protein content in milled rice (%)	Value of mido (taste)	Sensory attributes				
			Overall acceptability	Flavor	Stickiness	Hardness	Colar and gloss
Koshihikari	5.5	73.8	−0.03	−0.04	−0.05	−0.01	−0.08
SCM1+3+4	5.4	75.3	−0.13	−0.06	0.00	0.06	−0.09

Sakura price was selected from the pyramiding line SCM1+3+4.

Table S4. Culm and panicle length.

		Culm length (cm)			Panicle length (cm)		
Parents	Koshihikari	100.7	(100)		20.1	(100)	
	Habataki	84.3	(84)	***	25.7	(128)	***
	Chugoku 117	93.7	(93)	***	19.1	(95)	
1 QTL	SCM1	98.9	(98)		20.1	(100)	
	SCM2	101.4	(101)		20.9	(104)	
	SCM3	98.3	(98)		20.3	(101)	
	SCM4	100.9	(100)		20.4	(102)	
2 QTLs	SCM1+2	101.0	(100)		21.4	(107)	***
	SCM1+3	98.9	(98)		21.4	(107)	***
	SCM1+4	98.5	(98)		19.6	(98)	
	SCM2+3	101.3	(101)		21.9	(109)	***
	SCM2+4	99.0	(98)		20.7	(103)	
	SCM3+4	100.5	(100)		20.8	(104)	
3 QTLs	SCM1+2+3	98.1	(97)		21.4	(107)	***
	SCM1+2+4	99.5	(99)		20.8	(104)	
	SCM1+3+4	102.5	(102)		21.4	(107)	***
	SCM2+3+4	103.0	(102)		21.9	(109)	***
4 QTLs	SCM1+2+3+4	101.6	(101)		21.4	(107)	***

*, ** and *** indicate significant differences compared to Koshihikari at the 5%, 1% and 0.1% level (Dunnett's test). The numbers in parentheses show the values relative to Koshihikari (100).

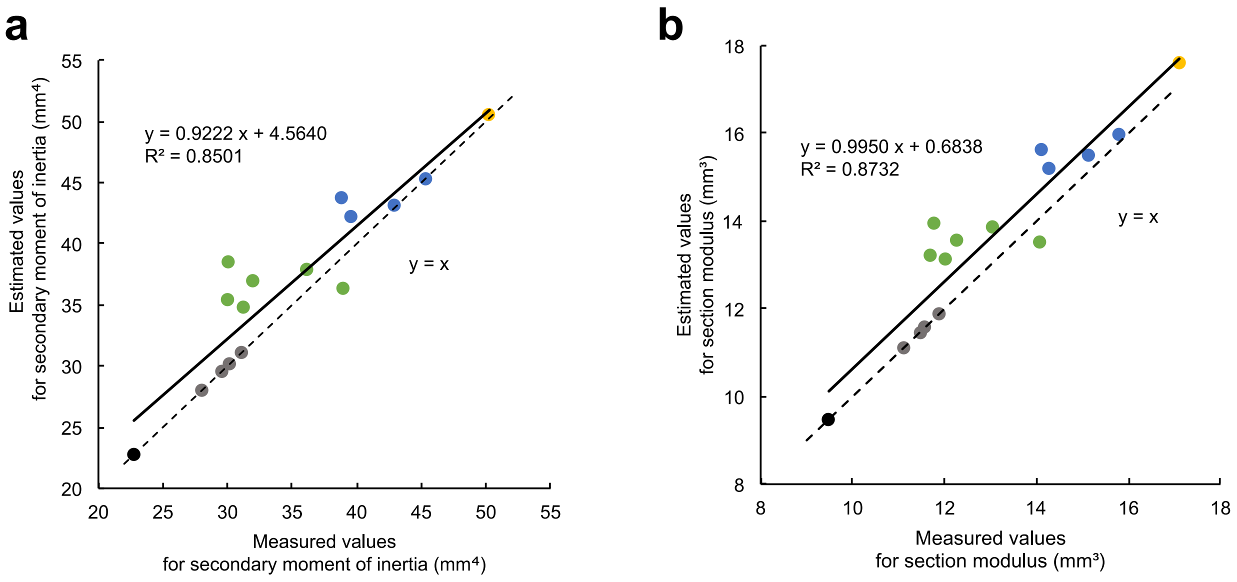

Fig. S1. The relationship between the estimated and measured secondary moment of inertia and section modulus excluding interactions for Koshihikari and pyramiding lines. (**a**) For the secondary moment of inertia, the regression equation is y = 0.922 x + 4.564 with a coefficient of determination of 0.850. (**b**) For the section modulus, the regression equation is y = 0.995 x + 0.684 with a coefficient of determination of 0.873. Dashed line indicates y = x (m = 1).

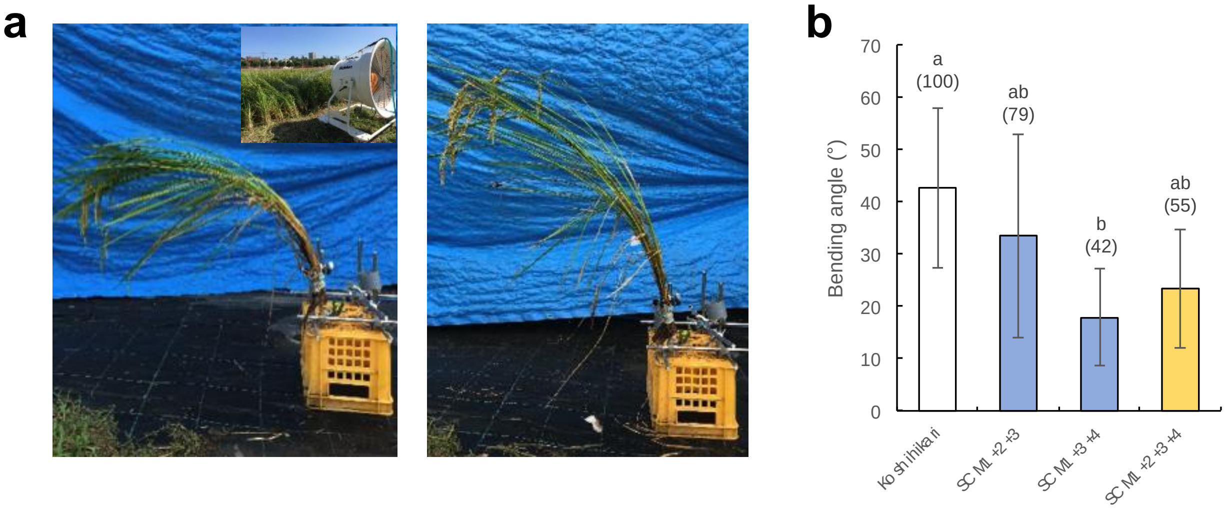

Fig. S2. Evaluation of lodging resistance using an artificial typhoon test.

(a) Representative photographs of Koshihikari (left) and SCM1+3+4 (right) after the artificial typhoon test. (b) Bending angle of culm; each column shows mean ± SD (n = 3). Different letters indicate significant differences between lines at the 5% level (Tukey's test). The numbers in parentheses show the values relative to Koshihikari (100).
